# Distribution of lipid droplets in hippocampal neurons and microglia: impact of diabetes and exercise

**DOI:** 10.26508/lsa.202302239

**Published:** 2024-08-08

**Authors:** Gezime Seferi, Harald S Mjønes, Mona Havik, Herman Reiersen, Knut Tomas Dalen, Kaja Nordengen, Cecilie Morland

**Affiliations:** 1 Section for Pharmacology and Pharmaceutical Biosciences, Department of Pharmacy, Faculty of Mathematics and Natural Sciences, University of Oslo , Oslo, Norway; 2 Institute of Basic Medical Sciences, Faculty of Medicine, University of Oslo , Oslo, Norway; 3 Department of Neurology, Oslo University Hospital, Oslo, Norway

## Abstract

Neuroinflammation, aging, and neurodegenerative disorders are associated with excessive accumulation of neutral lipids in lipid droplets (LDs) in microglia. Type 2 diabetes mellitus (T2DM) may cause neuroinflammation and is a risk factor for neurodegenerative disorders. Here, we show that hippocampal pyramidal neurons contain smaller, more abundant LDs than their neighboring microglia. The density of LDs varied between pyramidal cells in adjacent subregions, with CA3 neurons containing more LDs than CA1 neurons. Within the CA3 region, a gradual increase in the LD content along the pyramidal layer from the hilus toward CA2 was observed. Interestingly, the high neuronal LD content correlated with less ramified microglial morphotypes. Using the *db/db* model of T2DM, we demonstrated that diabetes increased the number of LDs per microglial cell without affecting the neuronal LD density. High-intensity interval exercise induced smaller changes in the number of LDs in microglia but was not sufficient to counteract the diabetes-induced changes in LD accumulation. The changes observed in response to T2DM may contribute to the cerebral effects of T2DM and provide a mechanistic link between T2DM and neurodegenerative disorders.

## Abstract (precis)

Lipid droplets (LDs) are differently distributed in the CA1 and CA3 of the mouse hippocampus, and even along the CA3. Diabetes increased microglial LDs, suggesting a neurodegenerative mechanism, which is modestly affected by high-intensity exercise.

## Introduction

Lipid homeostasis is necessary for maintaining neural function and brain plasticity (1), and dysregulated lipid metabolism is a characteristic of brain aging and age-related neurodegenerative diseases (2, 3, 4). Despite being the second most lipid-rich organ, the common dogma is that the brain has an exceptionally low capacity for storage and oxidation of lipids for energy production. Lipids can be divided into two main classes: neutral lipids, mainly consisting of triacylglycerols (TAGs), cholesteryl esters (CEs), retinyl esters, wax esters, and terpenes; and polar lipids, such as phospholipids, glycolipids, and sphingolipids. Accumulation of lipids in the brain (named “lipid bodies,” “lipid saccules,” “lipid inclusions,” etc.) was reported decades ago (5, 6), but their roles in brain function and brain disorders have largely been overlooked by the research community until recently. Recent advances in the field have revealed increased densities of lipid droplets (LDs) in neurodegenerative disorders (7, 8). LDs consist of a core of neutral lipids, predominantly TAGs and CEs, captured within a monolayer of amphipathic phospholipids and various LD-binding proteins. In addition to serving as a reservoir for fuel during periods of nutrient deprivation or cellular stress in most cells, the lipids stored in LDs may be used in membrane formation and remodeling, lipoprotein trafficking, and buffering of toxic lipids, and as a source of inflammation regulators (for instance, arachidonic acid and its metabolites). Based on the low capacity for beta-oxidation in most brain cells, non-metabolic roles of LDs appear more likely.

In the brain, LDs have mainly been described in hippocampal microglia where they seem to accumulate during aging (9, 10, 11) and age-related neurodegenerative disorders (8, 9, 12). LDs have also been observed in astrocytes (13) and specific types of neurons (14, 15). In neuronal cultures, the LD content is enhanced by cellular stress, such as excitotoxicity, oxidative stress, and exposure to elevated levels of fatty acids (7, 16). High levels of circulating free fatty acids, reduced ability for glucose disposal, and enhanced insulin resistance are hallmarks of obesity and type 2 diabetes mellitus (T2DM). The same goes for the accumulation of lipids and toxic lipid products in non-adipose tissues. A strong association has been proposed between T2DM and cognitive impairment, Alzheimer’s disease (AD), and other dementias (17, 18, 19, 20, 21, 22). It is not known whether LDs in the brain are regulated in response to fluctuations in circulating levels of lipids or whether ectopic accumulation of LDs in brain cells represents a mechanistic link between T2DM and dementia.

Exercise is highly beneficial for people with T2DM as it counteracts many of the causes and consequences of T2DM by reducing overweight, ectopic lipid accumulation, and insulin resistance in skeletal muscle and adipose tissues (23, 24). Exercise is also beneficial for the brain through a combination of indirect and direct effects. Although exercise may affect LD accumulation in peripheral tissues, effects on LDs in the brain have not been reported. Furthermore, the impact of intracellular (dys)regulation of LDs and/or dysregulated distribution of LDs between brain cells on the overall homeostasis and function of the brain remains unclear.

**Figure 1.**
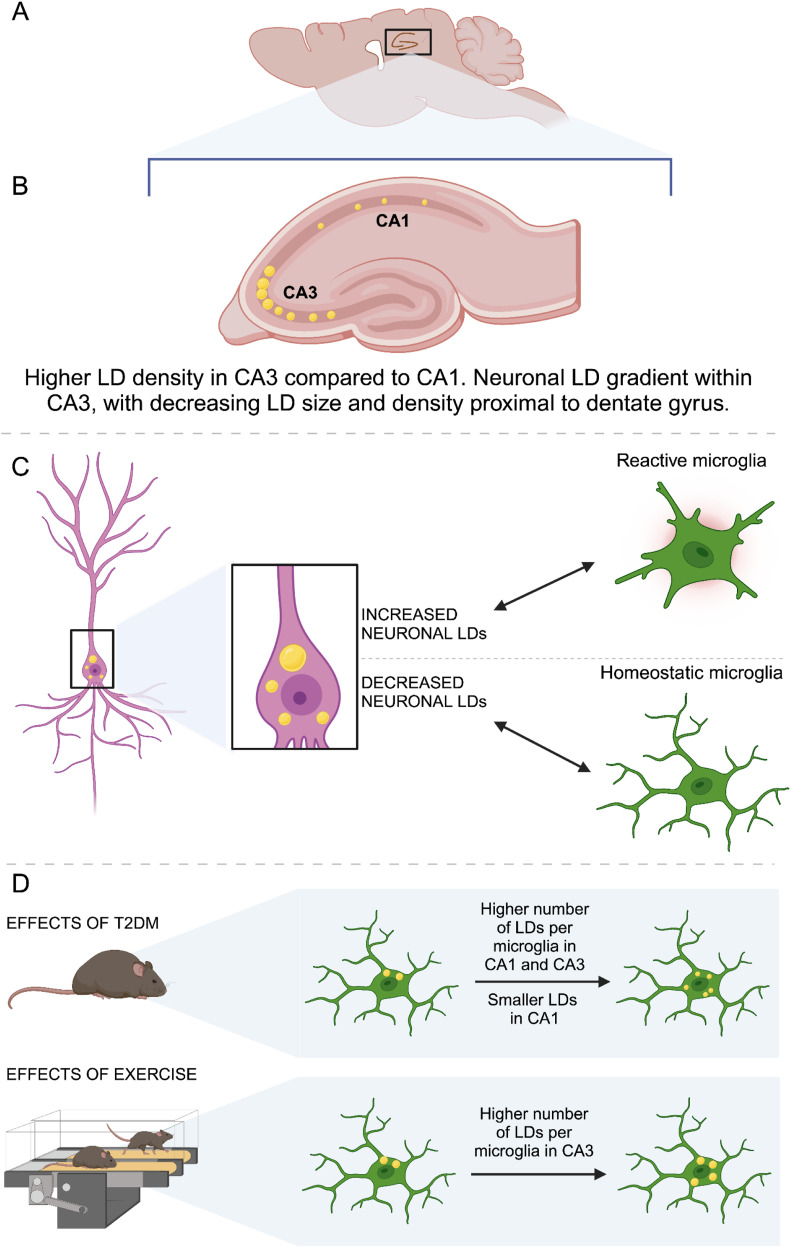
Overview of the results. **(A)** Parasagittal sections of the mouse brain were used for the analysis of microglia and lipid droplets (LDs). **(B)** LDs were detected in neurons and microglia in the cornu ammonis 1 (CA1) and cornu ammonis 3 (CA3) of the hippocampus. The density of LDs was higher in the CA3 than in the CA1, and there was a gradient of neuronal LDs along the CA3, with a lower density proximal to the dentate gyrus (DG) and a higher density proximal to the CA2. **(C)** In the CA1, high neuronal LD density was associated with more reactive microglial morphotypes, whereas in the CA3, larger neuronal LDs were associated with less reactive microglial morphotypes. **(D)** In response to the obese/diabetic phenotype, the *db/db* showed a higher number of LDs per microglial cell. In the CA1, but not in the CA3, microglia of obese/diabetic *db/db* mice had smaller LDs compared with their non-diabetic *db/*^*+*^ littermates. The neuronal LD density and size were unaffected by the obese/diabetic phenotype. Both *db/db* mice and *db/*^*+*^ littermates were exposed to high-intensity interval exercise for 8 wk. Neither the number nor the size of LDs in neurons was affected by exercise, but the number of LDs per microglial cell in the CA3 was higher in exercised mice than in their sedentary littermates.

In the present study, we investigated the presence of LDs in neurons, astrocytes, and microglia in the hippocampal formation. We further explored whether LDs distribute differently between the pyramidal neurons of the cornu ammonis 1 (CA1) and cornu ammonis 3 (CA3), and the granule neurons of the dentate gyrus (DG), as well as within the subregions of these areas. At the cellular level, we characterized the distribution of LDs between microglia and neurons in the same subregions. Furthermore, we investigated whether the neuronal or microglial LD content correlated with a shift in microglial morphology. To study whether the accumulation of LDs in the brain was affected by high levels of circulation lipids, we analyzed the LD density and size in neurons and microglia in the hippocampus of the *db/db* mouse model of T2DM and compared it with what was found in non-diabetic *db/*^*+*^ littermates. The former is known to have increased circulating levels of lipids, which is also a hallmark of human T2DM. Finally, to reveal whether exercise affects LD accumulation in the brain, LD analysis was performed in the hippocampus of *db/db* and the *db/*^*+*^ mice that remained sedentary or were exposed to 8 wk of high-intensity interval training (HIIT). The main findings are summarized in Fig 1A–D.

## Results

To determine whether LDs are present in neurons, astrocytes, and/or microglia of the hippocampus of adult control (*db/*^*+*^) mice, we performed a series of labeling experiments where brain sections were costained with the neutral lipid marker BODIPY 493/503, the microglial marker Iba1, the neuronal marker NeuroTrace, or the astrocyte marker GFAP in whole-brain parasagittal sections. The ability of BODIPY to selectively identify LDs is well documented in the literature, by labeling with antibodies against LD-associated proteins, stimulated Raman scattering microscopy, or coherent anti-Stokes Raman scattering (10, 25, 26).

### LDs are present in microglia and neurons in the cornu ammonis

**Figure 2.**
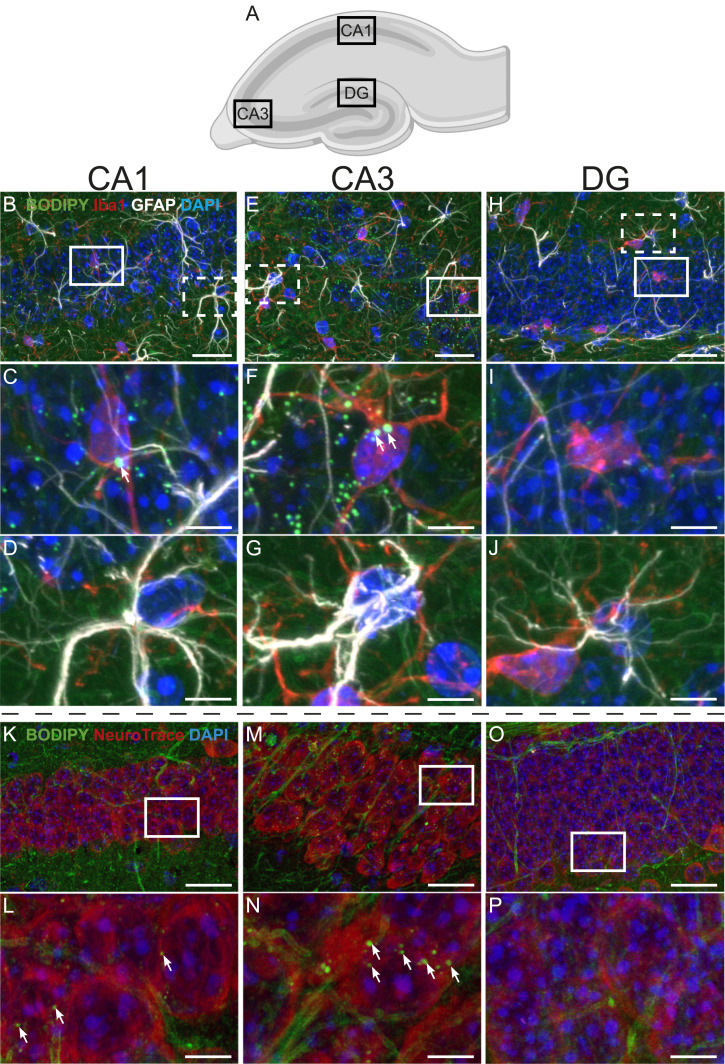
Lipid droplets in hippocampal microglia and neurons. **(A)** Cartoon of the mouse hippocampus, with the regions cornu ammonis 1 (CA1), cornu ammonis 3 (CA3), and DG outlined. **(B, C, D, E, F, G, H, I, J)** Maximum intensity projections of confocal Z-stack images of the hippocampus of a sedentary db^/+^ (control) mouse labeled for the microglial marker Iba1 (red), the astrocyte marker GFAP (white), the DNA marker DAPI (blue), and the LD marker BODIPY 493/503 (green). **(B)** CA1 pyramidal cell layer. **(C)** White rectangle identifies a microglial cell (red) with LDs (green dots), which is shown at higher magnification in (C). Note the large LD (a green dot indicated by a white arrow) within the microglial soma and the presence of smaller LDs outside the microglia, presumably localized in neurons. **(B, D)** Dotted rectangle in (B) identifies an astrocyte (white) with no LDs, which is shown in higher magnification in (D). **(E)** CA3 pyramidal cell layer. **(F)** White rectangle identifies a microglial cell (red) with LDs (green dots), which is shown at higher magnification in (F). Note the two large LDs (green dots indicated by white arrows) within the microglial soma and the high density of smaller LDs outside the microglia. **(E, G)** Dotted rectangle in (E) identifies an astrocyte (white) with no LDs, which is shown in higher magnification in (G). **(H)** DG granule cell layer. **(I)** White rectangle identifies a microglial cell (red) with no LDs, which is shown at higher magnification in (I). **(H, J)** Dotted rectangle in (H) identifies an astrocyte (white) with no LDs, which is shown in higher magnification in (J). **(K, L, M, N, O, P)** Confocal Z-stack images of the hippocampus of a sedentary db/^+^ (control) mouse labeled with the neuronal marker NeuroTrace (red), BODIPY 493/503 (green), and DAPI (blue). **(K)** CA1 pyramidal cell layer. **(L)** White rectangle identifies a few pyramidal neurons (red) with LDs (green dots), which are shown at higher magnification in (L). Note the small LDs localized within the neurons (green; some are indicated by white arrows). **(M)** CA3 pyramidal cell layer. **(N)** White rectangle identifies a few pyramidal neurons (red) with LDs (green dots), which are shown at higher magnification in (N). Note the high density of small LDs localized within the neurons (green; some are indicated by white arrows). **(O, P)** DG granule cell layer. The white rectangle identifies a few granule neurons (red) with no LDs, which are shown at higher magnification in (P). Scale bars: 20 μm in (B, C, D, K, L, M, N, O) and 5 μm in (C, D, E, F, G, H, I, J, L, M, N, O, P).

LDs were observed in the hippocampal formation (Fig 2A). After pixel classification of each optical section in the Z-stack and further processing in Fiji (ImageJ, v.2.1.0), LDs were found in microglia and neurons, but no LDs were observed in astrocytes (Fig 2). Therefore, detailed studies of LDs were performed in microglia and neurons in the hippocampus proper. Within the hippocampus, confocal microscopy revealed that LDs were distributed unequally between the different microglial and neuronal populations; LDs were observed in microglia within the pyramidal cell layer and the stratum radiatum of the CA1 (Fig 2B and C) and the CA3 (Fig 2E and F), but not in the DG (Fig 2H–J). Similarly, pyramidal neurons of the CA1 (Fig 2K and L) and the CA3 (Fig 2M and N) showed high densities of LDs, whereas LDs were observed to a lesser extent in granule neurons of the DG (Fig 2O and P).

**Figure 3.**
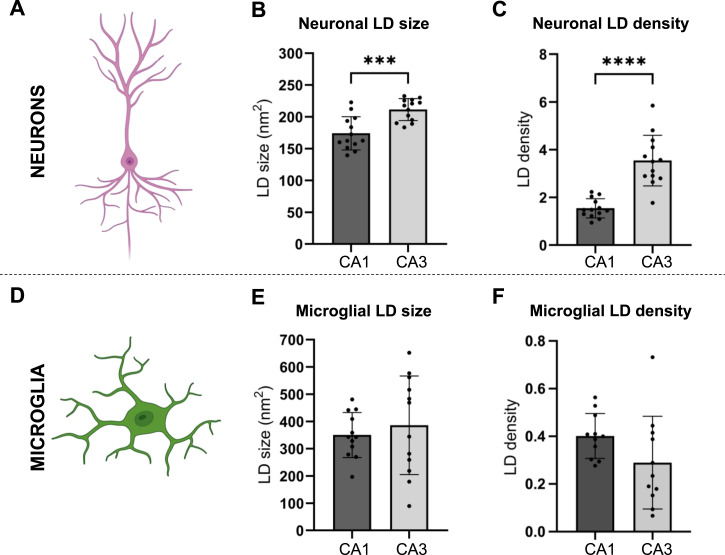
Regional differences in LD accumulation in the hippocampus. **(A)** Cartoon of a neuron. **(B, C)** Bar plots. Bars show the mean values from the cornu ammonis 1 (*CA1*; dark gray) *and* cornu ammonis 3 (*CA3*; light gray). Error bars indicate the SD. The dots represent the mean measure from one individual animal. **(B)** LD size in neurons in the CA1 and the CA3. **(C)** LD density in neurons in the CA1 and the CA3. **(D)** Cartoon of a microglial cell. **(E, F)** Bar plots. Bars show the mean values from the cornu ammonis 1 (*CA1*; dark gray) *and* cornu ammonis 3 (*CA3*; light gray). Error bars indicate the SD. The dots represent the mean measure from one individual animal. **(E)** LD size in microglia in the CA1 and the CA3. **(F)** LD density in microglia in the CA1 and the CA3. Number of animals (n): (B, C):13; (E):12; (F):11. ****P ≤ 0.0001.

Interestingly, quantitative measurements of the LDs in neurons (Fig 3A–C) revealed that the mean size of LDs in neurons was 18% smaller in the CA1 (174.1 ± 26.1 nm^2^, mean ± SD) than in the CA3 (211.4 ± 17.3 nm^2^; *P* = 0.0003, paired *t* test) (Fig 2K and L versus Fig 2M and N; quantified in Fig 3B). The smaller mean LD size was accompanied by a 56% lower LD density in the CA1 pyramidal neurons (1.55 ± 0.40 LDs/10 μm^2^, mean ± SD) compared with the CA3 pyramidal neurons (3.55 ± 1.06 LDs/10 μm^2^; *P* < 0.0001, paired *t* test) (Fig 2K and L versus Fig 2M and N; quantified in Fig 3C), indicating a substantially lower LD content of the CA1 pyramidal neurons compared with the CA3 pyramidal neurons. Quantitative measurements of LDs in microglia in the CA1 and the CA3 (Fig 3D–F) did not differ in size (350.4 ± 82.7 nm^2^ in the CA1 versus 386.2 ± 180.6 nm^2^ in the CA3, mean ± SD) (Fig 2B and C versus Fig 2E and F; quantified in Fig 3E) nor in density (0.402 ± 0.094 LDs/10 μm^2^ in the CA1 versus 0.288 ± 0.194 LDs/10 μm^2^ in the CA3, mean ± SD) (Fig 2B and C versus Fig 2E and F; quantified in Fig 3F). The analyses presented in Fig 3 also revealed that the LD density was 3.8-fold higher in neurons compared with microglia located in the CA1 and 12.3-fold higher in neurons compared with microglia located in the CA3, mainly reflecting the difference in neuronal LDs. On average, the neuron:microglial size ratio of LDs in the CA1 region was 0.55 ± 0.21 (mean ± SD), and in the CA3 region, this ratio was 0.74 ± 0.52 (mean ± SD), demonstrating that LDs in microglia were larger in size than the neuronal LDs.

### LDs are distributed unequally between pyramidal neurons in different subregions of the CA3

**Figure 4.**
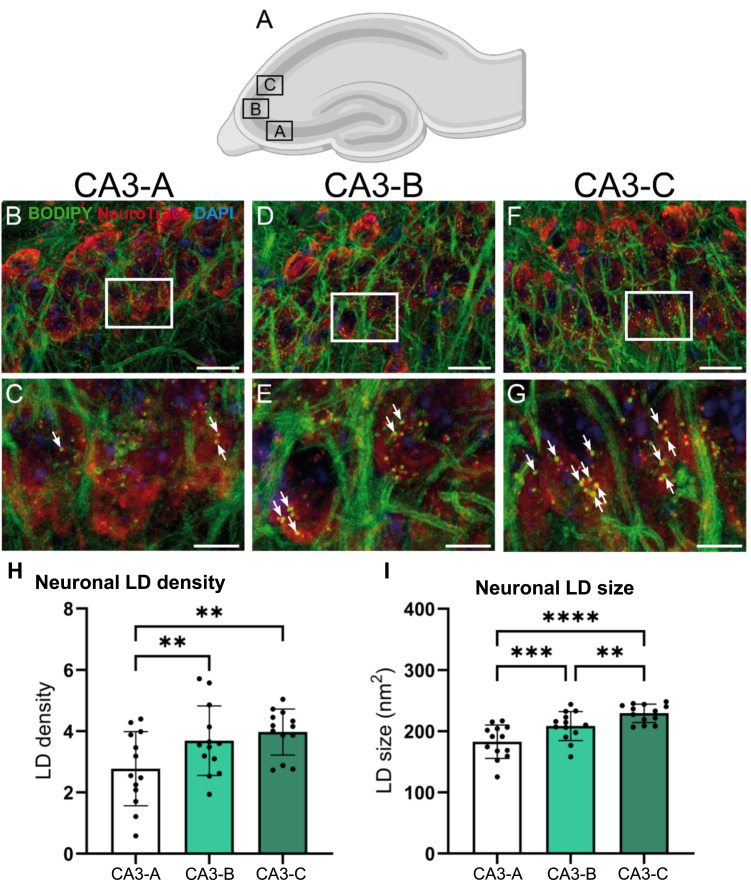
Regional differences in neuronal LD accumulation between the CA3 subfields. **(A)** Cartoon of the mouse hippocampus, with the subfields cornu ammonis 3 (CA3)-A, CA3-B, CA3-C outlined. **(B, C, D, E, F, G)** Maximum intensity projections of confocal Z-stack images of the hippocampus of a sedentary *db/*^*+*^ (control) mouse stained with the neuronal marker NeuroTrace (red), the LD marker BODIPY 493/503 (green), and the nucleus marker DAPI (blue). Some LDs are indicated by arrows. **(B)** CA3-A. **(C)** White rectangle identifies a few pyramidal neurons (red) with LDs (green dots), which are shown at higher magnification in (C). **(D)** CA3-B. **(E)** White rectangle identifies a few pyramidal neurons (red) with LDs (green dots), which are shown at higher magnification in (E). **(F)** CA3-C. **(G)** White rectangle identifies a few pyramidal neurons (red) with LDs (green dots), which are shown at higher magnification in (G). **(H, I)** Bar plots. Bars show the mean of the CA3-A (white), CA3-B (light green), or CA3-C (dark green); error bars indicate the SD. The dots represent the mean measure of one individual mouse. **(H)** Comparison of the mean LD density in neurons between CA3 subfields. **(I)** Comparison of the mean LD size in neurons between the CA3 subfields. Number of animals (n): (H):*13*; (I):*13*. **P ≤ 0.010; ***P ≤ 0.0010; ****P ≤ 0.0001. Scale bars: 20 μm in (B, D, F) and 5 μm in (C, E, G).

LDs did not accumulate uniformly within the CA3 region. Images were taken at three locations along the pyramidal cell layer (Fig 4A). Moving along the pyramidal layer from the DG toward the CA2, there was a gradual increase in the density of LDs from 2.78 ± 1.21 LDs/10 μm^2^ in the part closest to the DG (Fig 4B and C; quantified in Fig 4H) to 3.69 ± 1.13 LDs/10 μm^2^ in the middle part (32% higher; *P* = 0.002, repeated-measures one-way ANOVA and Tukey’s post hoc test) (Fig 4D and E; quantified in Fig 4H). In the pyramidal cells closest to the CA2, the LD density was 3.97 ± 0.75 LDs/10 μm^2^, which was 43% higher than in the part closer to the DG (*P* = 0.0076, repeated-measures one-way ANOVA and Tukey’s post hoc test) (Fig 4F and G; quantified in Fig 4H). Similarly, a gradient in the size of the LDs was observed in the pyramidal neurons: LD sizes were 182.81 ± 27.34 nm^2^ (mean ± SD) in the CA3 closest to the DG (Fig 4B and C; quantified in Fig 4I), 208.52 ± 23.64 μm^2^ in the middle part of the CA3 (14% larger; *P* = 0.0003) (Fig 4D and E; quantified in Fig 4I), and 229.44 ± 15.06 μm^2^ in the part closer to the CA2 (26% larger than in the part closer to the DG [*P* < 0.0001] and 10% larger than in the middle part [*P* = 0.0014, repeated-measures one-way ANOVA and Tukey’s post hoc test]) (Fig 4F and G; quantified in Fig 4I).

### Association between the LD content in microglia or neurons and the microglial morphotype

**Figure 5.**
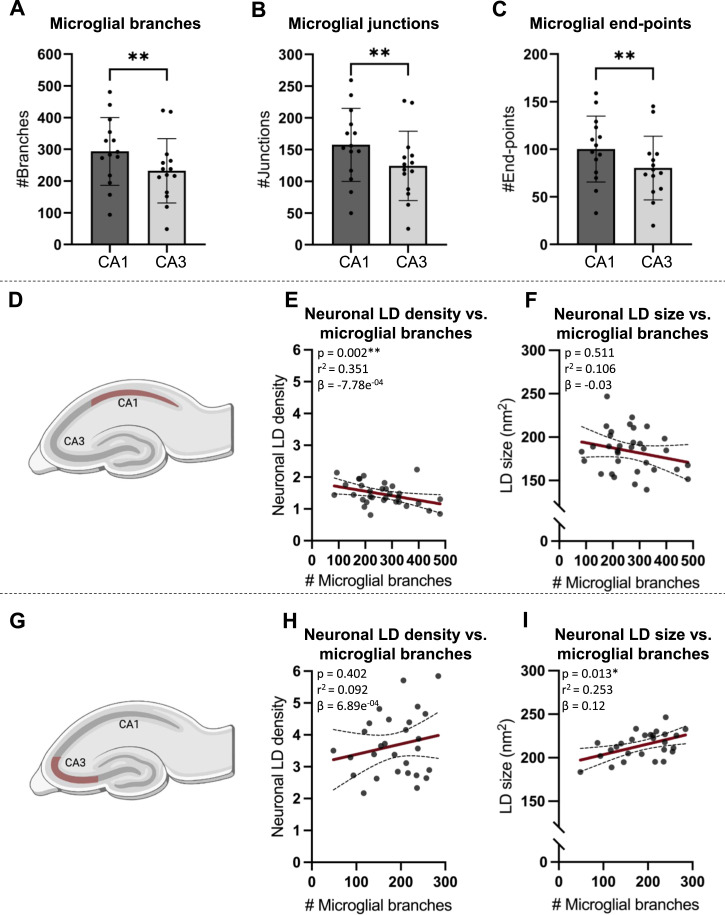
Microglial ramifications and correlation between neural lipid droplets and microglial ramifications. **(A, B, C)** Bar plots. Bars represent the mean value of measurements from the CA1 (dark gray) and the CA3 (light gray). Error bars indicate the SD. The dots represent the mean measure from one individual animal. Microglial reactivity was quantified as the mean number of branches per cell (#Branches), the mean number of junctions per cell (#Junctions), and the mean number of end-point voxels (#End-points). Analyses were performed for all animals combined and corrected for genotype and intervention. **(D)** Cartoon of the hippocampus where the CA1 is highlighted in red to illustrate that the graphs in E and F are from this subregion. **(E, F)** Scatter plots. Each dot represents the mean measure from one animal, and the solid line represents the trendline, for which Pearson’s R-value (r2) is given above each plot along with the *P*-value for the correlation. The shaded area represents the SD. **(E)** Correlation between the density of LDs in neurons and the number of branches per microglial cell in the CA1 (square-root–transformed data). **(F)** Correlation between the size of LDs in neurons and the number of branches per microglial cell in the CA1 (untransformed data). **(G, H, I)** Cartoon of the hippocampus where the CA3 is highlighted in red to illustrate that the graphs in (H, I) are from this subregion. **(H, I)** Scatter plots. Each dot represents the mean measure from one animal, and the solid line represents the trendline, for which Pearson’s R-value (r2) is given above each plot along with the *P*-value for the correlation. The shaded area represents the SD. **(H)** Correlation between the density of LDs in neurons and the number of branches per microglial cell in the CA3 (square-root–transformed data). **(I)** Correlation between the size of LDs in neurons and the number of branches per microglial cell in the CA3 (untransformed data). Number of animals (n): (A, B, C): CA1, n = 14; CA3, n = 14; (E): n = 32; (F): n = 33; (H): n = 28; (I): n = 27. *P ≤ 0.05.

To determine whether the regional higher density and larger size of LDs in the CA3 (Fig 5D) compared with the CA1 pyramidal layer (Fig 5G) were accompanied by microglia with a less ramified morphotype, the mean number of branches, junctions, and end-point voxels per microglial cell for each animal was determined from segmented images. Microglia in a more reactive state show the reduced numbers of ramifications compared with homeostatic microglia; therefore, a low number of branches, junctions, and end-point voxels were used as proxies for microglial reactivity. A comparison of the mean number of branches per microglial cell in the CA1 and the CA3 within the same animal revealed that the microglia residing in the CA1 region had a significantly higher number of branches than the microglia in the CA3 (CA1: 294 ± 107 branches/cell versus the CA3: 232 ± 101 branches/cell; *P* = 0.0085, paired *t* test) (Fig 5A). Accordingly, the mean number of junctions (CA1: 158 ± 57.6 junctions/cell versus CA3: 124 ± 54.7 junctions/cell; *P* = 0.0086, paired *t* test) (Fig 5B) and the mean number of end-point voxels (CA1: 100 ± 34.7 end-points/cell versus CA3: 80.3 ± 33.4 end-points/cell; *P* = 0.006, paired *t* test) (Fig 5C) were higher in the CA1 compared with the CA3.

Furthermore, linear regression analyses were used to investigate whether the degree of microglial ramification correlated with the density or size of LDs in neurons. In the CA1, the density of LDs (% of the neuronal area covered by LDs) in neurons showed a negative correlation with the number of microglial branches (Fig 5E; *P* = 0.002, r^2^ = 0.351, β = −7.78 × 10^−04^), suggesting a higher density of neuronal LDs in animals with more reactive microglia. The mean size of the LDs in neurons showed no correlation with the number of microglial branches (Fig 5F). Similarly, the density of LDs in neurons in the CA3 region did not correlate with the number of microglial branches (Fig 5H), whereas the mean size of LDs in neurons in the CA3 region (Fig 5I) showed a positive correlation with the number of microglial branches (*P* = 0.013, r^2^ = 0.253, β = 0.12). Hence, small LD size in neurons in the CA3 region correlated with a reactive microglial morphotype.

### Effects of diabetes and exercise on LDs in neurons

**Figure 6.**
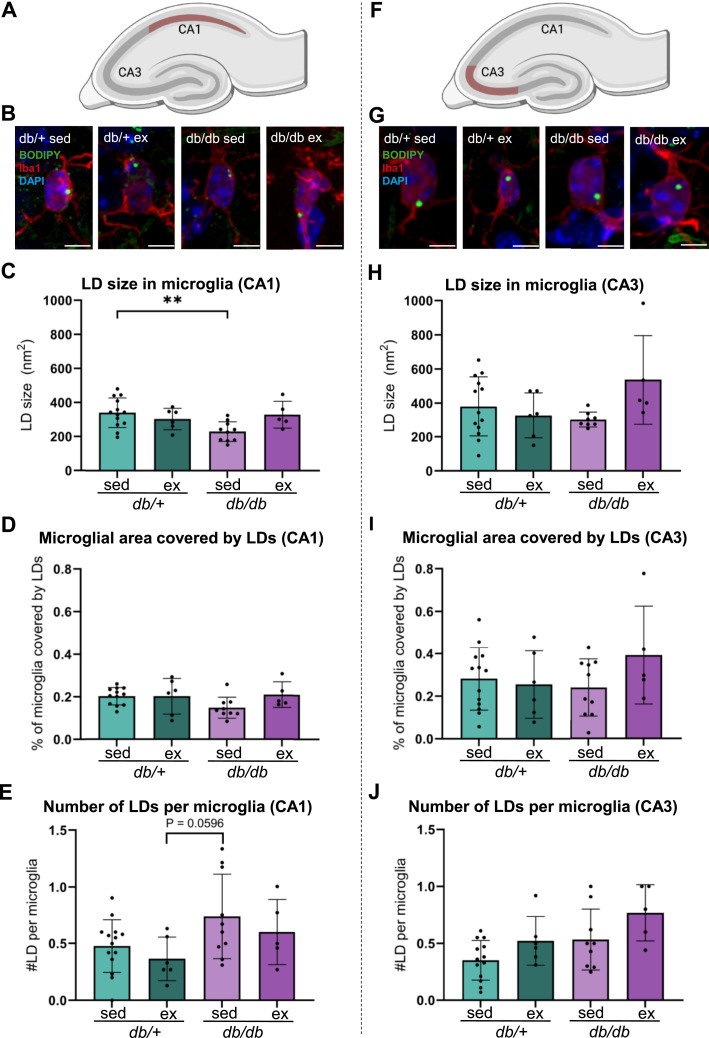
Effects of exercise on hippocampal lipid droplets in microglia in diabetic and non-diabetic mice. **(A)** Cartoon of the mouse hippocampus, with the CA1 region outlined in red. **(B)** Maximum intensity projections of confocal Z-stack images of microglia from sedentary and exercised *db/*^*+*^ and *db/db* mice labeled for the microglial marker Iba1 (red), the LD marker BODIPY 493/503 (green), and the nucleus marker DAPI (blue). **(C, D, E)** Bar plots. Bars show the mean of measurements from the CA1 region for sedentary *db/*^*+*^ mice (*db/*^*+*^ sed; light green), exercised *db/*^*+*^ mice (*db/*^*+*^ ex; dark green), sedentary *db/db* mice (*db/db* sed; light purple), and exercised *db/db* mice (*db/db* ex; dark purple). Error bars indicate the SD. The dots represent the mean measure from each animal. **(C)** Microglial LD size (nm^2^) in the CA1. **(D)** Percentage of microglial area covered by LDs in the CA1. **(E)** Number of LDs per microglial cell in the CA1. **(F)** Cartoon of the mouse hippocampus, with the CA3 region outlined in red. **(G)** Maximum intensity projections of confocal Z-stack images of microglia from sedentary and exercised *db/*^*+*^ and *db/db* mice labeled for the microglial marker Iba1 (red), the LD marker BODIPY 493/503 (green), and the nucleus marker DAPI (blue). **(H, I, J)** Bar plots. Bars show the mean of measurements from the CA3 region for sedentary *db/*^*+*^ mice (*db/*^*+*^ sed; light green), exercised *db/*^*+*^ mice (*db/*^*+*^ ex; dark green), sedentary *db/db* mice (*db/db* sed; light purple), and exercised *db/db* mice (*db/db* ex; dark purple). Error bars indicate the SD. The dots represent the mean measure from each animal. **(H)** Microglial LD size (nm^2^) in the CA3. **(I)** Percentage of microglial area covered by LDs in the CA3. **(J)** Number of LDs per microglial cell in the CA3. Number of animals (n) for *db/+; db/db*; sedentary; exercised mice used in each analysis: (C): n= 13; 6; 10; 5. (D): n= 12; 6; 9; 5. (E): n= 14; 6; 10; 5. (H): n= 13; 6; 8; 5. (I): n= 13; 6; 10; 5. (J): n= 13; 6; 9; 5. **P ≤ 0.01. Scale bars: 5 μm.

**Figure S1.**
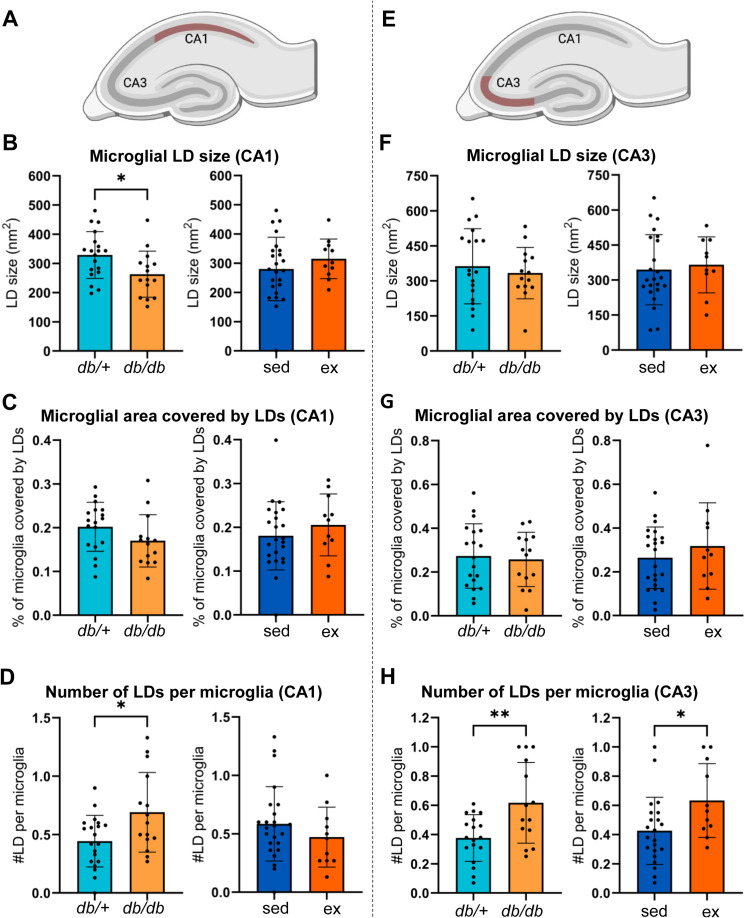
Effects of genotype and exercise on hippocampal lipid droplets in microglia. **(A)** Cartoon of the mouse hippocampus, with the CA1 region outlined in red. **(B, C, D)** Bar plots. Bars show the mean of measurements from the microglia of the CA1 region for *db/*^*+*^ mice (*db/*^*+*^; turquoise) and *db/db* mice (*db/db*; orange) regardless of exercise, or sedentary mice (sed; blue) and exercised mice (ex; dark orange) regardless of genotype. Error bars indicate the SD. The dots represent the mean measure from each animal. **(B)** Microglial LD size (nm^2^) in the CA1. **(C)** Percentage of microglial area covered by LDs in the CA1. **(D)** Number of LDs per microglial cell in the CA1. **(E)** Cartoon of the mouse hippocampus, with the CA3 region outlined in red. **(F, G, H)** Bar plot. Bars show the mean of measurements from the microglia of the CA3 region for *db/*^*+*^ mice (*db/+*; turquoise) and *db/db* mice (*db/db*; orange) regardless of exercise, or sedentary mice (sed; blue) and exercised mice (ex; dark orange) regardless of genotype. Error bars indicate the SD. The dots represent the mean measure from each animal. **(F)** Microglial LD size (nm^2^) in the CA3. **(G)** Percentage of microglial area covered by LDs in the CA3. **(H)** Number of LDs per microglia in the CA3. Number of animals (n) for *db/+; db/db*; sedentary; exercised mice used in each analysis: (B): n= 19; 15; 24; 11. (C): n = 18; 14; 23; 11. (D): n= 20; 15; 24; 11. (F): n= 19; 14; 23; 10. (G): n = 19; 14; 23; 11. (H): n= 18; 14; 22; 11. *P ≤ 0.05; **P ≤ 0.01.

To determine whether a diabetic phenotype or the HIIT intervention affected the LD size and density in pyramidal neurons, the neuronal LDs were analyzed and compared between sedentary or exercised *db/*^*+*^ and *db/db* mice (Fig 6A, B, F, and G). In the CA1, the size of the LDs in microglia was lower in sedentary *db/db* mice than in sedentary *db/*^*+*^ mice (Fig 6C; 230.02 ± 57.40 nm^2^ in sedentary *db/db* mice versus 340.45 ± 86.92 nm^2^ in sedentary *db/*^*+*^ mice [*P* = 0.0099, two-way ANOVA and Tukey’s post hoc test]). In the same subregion, the percentage of the microglial area that was covered by LDs (Fig 6D) and the number of LDs per microglial cell (Fig 6E) were not different between the groups, but the number of LDs per microglial cell tended to be higher in sedentary *db/db* mice than in exercised *db/*^*+*^ mice (*P* = 0.060, two-way ANOVA and Tukey’s post hoc test). In the CA3, no statistically significant differences were detected between the groups, neither for the microglial LD size (Fig 6H), the percentage of microglial area that was covered by LDs (Fig 6I), nor the number of LDs per microglial cell (Fig 6J). Pooling the data and reanalyzing based on the genotype or intervention separately (Fig S1A–H) revealed that *db/db* mice, regardless of whether they were sedentary or exercised, showed a higher density (the number of LDs per microglial cell) in both the CA1 (Fig S1D; *db/*^*+*^: 0.444 ± 0.221 versus *db/db*: 0.691 ± 0.341 LDs/microglia; *P* = 0.0223, Welch’s *t* test) and the CA3 (Fig S1H; *db/*^*+*^: 0.377 ± 0.159 versus *db/db*: 0.617 ± 0.277 LDs/microglia; *P* = 0.009, Welch’s *t* test). In the CA1, each LD was smaller in size than in *db/*^*+*^ mice (Fig S1B; *db/*^*+*^: 329 ± 80.2 nm^2^ versus *db/db*: 263 ± 78.9 nm^2^; *P* = 0.023, Welch’s *t* test). Exercise, in general, did not result in large differences in the LD size or density in microglia, but in the CA3 region, a higher number of LDs per microglial cell were observed in response to exercise (Fig S1H; sedentary: 0.426 ± 0.230 versus exercised: 0.634 ± 0.253 LDs/microglia; *P* = 0.034, Welch’s *t* test).

**Figure 7.**
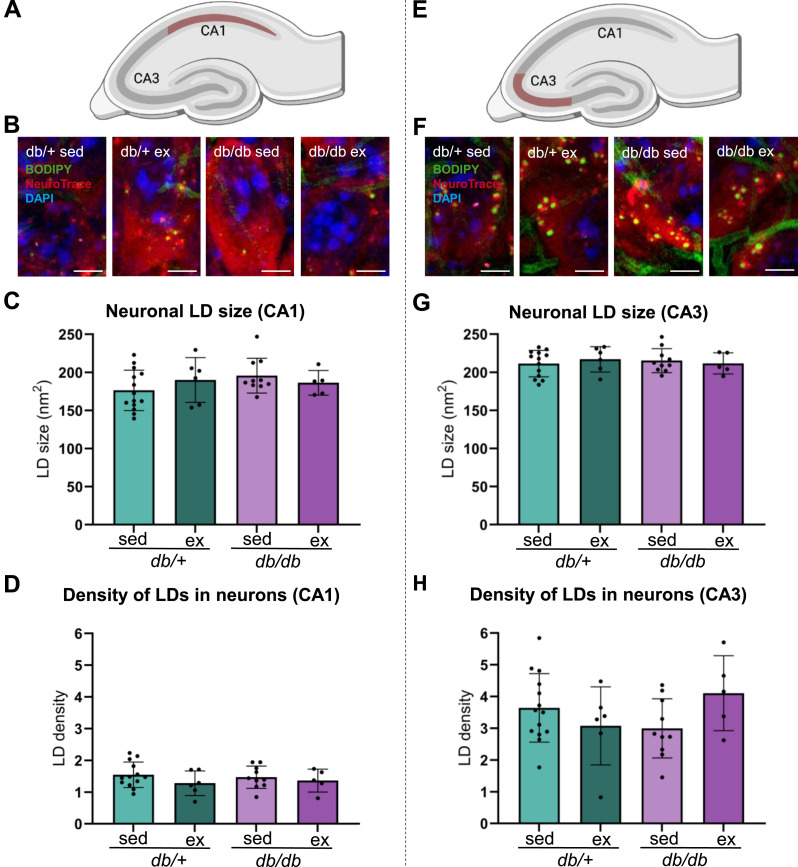
Effects of exercise on hippocampal lipid droplets in neurons in diabetic and non-diabetic mice. **(A)** Cartoon of the mouse hippocampus, with the CA1 region outlined in red. **(B)** Confocal Z-stack images of microglia from sedentary and exercised *db/*^*+*^ and *db/db* mice labeled for the microglial marker Iba1 (red), the LD marker BODIPY 493/503 (green), and the nucleus marker DAPI (blue). **(C, D)** Bar plots. Bars show the mean of measurements from the CA1 region for sedentary *db/*^*+*^ mice (*db/*^*+*^ sed; light green), exercised db/^+^ mice (*db/*^*+*^ ex; dark green), sedentary *db/db* mice (*db/db* sed; light purple), and exercised db/db mice (*db/*^*+*^ ex; dark purple). Error bars indicate the SD. The dots represent the mean measure from each animal. **(C)** Neuronal LD size in the CA1 across groups. **(D)** Neuronal LD density in the CA1 across groups. **(E)** Cartoon of the mouse hippocampus, with the CA3 region outlined in red. **(F)** Maximum intensity projections of confocal Z-stack images of neurons from sedentary and exercised *db/*^*+*^ and *db/db* mice labeled for the microglial marker Iba1 (red), the LD marker BODIPY 493/503 (green), and the nucleus marker DAPI (blue). **(G, H)** Bar plots. Bars show the mean of measurements from the CA1 region for sedentary *db/*^*+*^ mice (*db/*^*+*^ sed; light green), exercised *db/*^*+*^ mice (*db/*^*+*^ ex; dark green), sedentary *db/db* mice (*db/db* sed; light purple), and exercised *db/db* mice (*db/*^*+*^ ex; dark purple). Error bars indicate the SD. The dots represent the mean measure from each animal. **(G)** Neuronal LD size in the CA3 across groups. **(H)** Neuronal LD density in the CA3 across groups. Number of animals (n) for *db/+; db/db*; sedentary; exercised mice used in each analysis: (C): n= 14; 6; 10; 5. (D): n= 13; 6; 10; 5. (G): n= 13; 6; 10; 5. (H): n= 14; 6; 10; 5. Scale bars: 5 μm.

**Figure S2.**
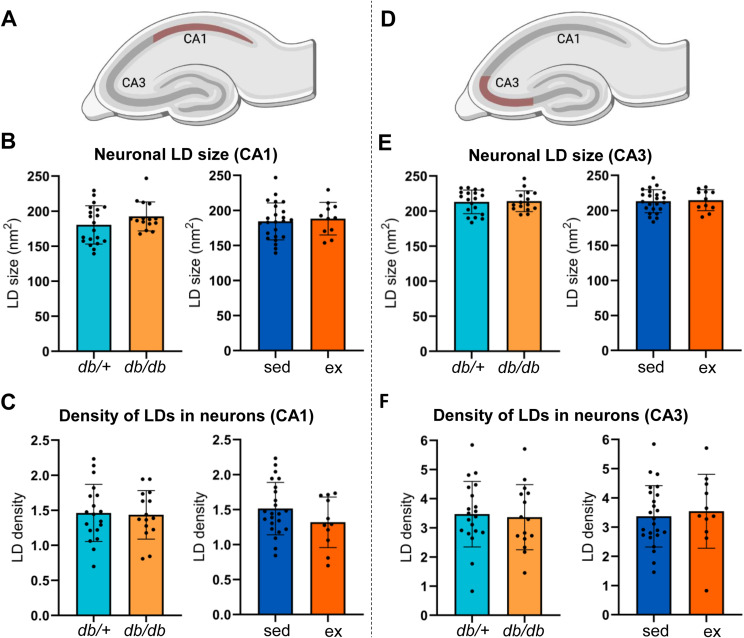
Effects of genotype and exercise on hippocampal lipid droplets in neurons. **(A)** Cartoon of the mouse hippocampus, with the CA1 region outlined in red. **(B, C)** Bar plot. Bars show the mean of measurements from the CA1 region for *db/*^*+*^ mice (*db/*+; turquoise) and *db/db* mice (*db/db*; orange) regardless of exercise, or sedentary mice (sed; blue) and exercised mice (ex; dark orange) regardless of genotype. Error bars indicate the SD. The dots represent the mean measure from each animal. **(B)** Neuronal LD size (nm^2^) in the CA1. **(C)** Density of LDs in the CA1 pyramidal neurons. **(D)** Cartoon of the mouse hippocampus, with the CA3 region outlined in red. **(E, F)** Bar plot. Bars show the mean of measurements from the CA3 region for *db/*^*+*^ mice (*db/+*; turquoise) and *db/db* mice (*db/db*; orange) regardless of exercise, or sedentary mice (sed; blue) and exercised mice (ex; dark orange) regardless of genotype. Error bars indicate the SD. The dots represent the mean measure from each animal. **(E)** Neuronal LD size (nm^2^) in the CA3. **(F)** Density of LDs in the CA3 pyramidal neurons. Number of animals (n) for *db/+; db/db*; sedentary; exercised mice used in each analysis: (B): n= 20; 15; 24; 11. (C): n= 19; 15; 23; 11. (E): n= 19; 15; 23; 11. (F): n= 20; 15; 24; 11.

The neuronal LD size (Fig 7C) and the density of LDs in neurons (Fig 7D) in the CA1 were not different between sedentary or exercised *db/*^*+*^ and *db/db* mice (Fig 7A, B, E, and F), nor were any differences observed in the CA3 (Fig 7G and H). Pooling the data and reanalyzing based on the genotype or intervention separately confirmed that the diabetic phenotype (*db/db*) did not affect the LD size or the density of LDs in neurons (Fig S2A–C for the CA1; Fig S2D–F for the CA3).

## Discussion

In the present study, we report that LDs are present in microglia and neurons of the hippocampus. We demonstrate a difference in the LD content (LD density and/or LD size) among subregions within the hippocampus. Microglia and neurons in, or close to, the pyramidal cell layer of the CA1 and the CA3 in the dorsal hippocampal formation showed high densities of LDs. We found a higher density of LDs in pyramidal neurons than in microglia in the hippocampus proper, but the neuronal LDs were smaller in size. The presence of LDs in microglia aligns with previous studies (10, 27). Some studies have also reported an astrocytic accumulation of LDs (13, 28, 29), but this was not observed in the present study (Fig 2D, G, and J). Most studies reporting LDs in astrocytes are, however, conducted in cultured astrocytes (30). Astrocytes have been reported to accumulate LDs if they are stressed, which will often be the case for astrocytes in culture (30). Previous studies reporting the presence of LDs in neurons are also mainly based on in vitro studies (31, 32, 33).

It is not understood why microglia and neurons of the hippocampus contain LDs. Various intrinsic or extrinsic events may lead to the accumulation of LDs in cells. For microglia, inflammation is a key trigger, and both treatment with LPS (10, 34) and increased ROS levels (11) stimulate LD accumulation. Furthermore, elevated concentrations of extracellular lipids and intracellular metabolic changes may also cause enhanced accumulation of LDs in microglia (35 *Preprint*). The presence and function of LDs in neurons are highly understudied. One study has reported neuronal accumulation of LDs in complex hereditary spastic paraplegia (HSP). This rare disease is characterized by weakness, spasticity of the lower limbs, and intellectual disability (15). A recessive form of HSP is caused by deleterious mutations in the *DDHD2* gene, which encodes the serine hydrolase DDHD2. The DDHD2 enzyme has been shown to be the principal TAG hydrolase in the mammalian brain (15). Interestingly, the same study found that genetic deletion, as well as pharmacological inhibition of this enzyme, led to massive LD accumulation in neurons and manifestation of HSP symptoms. Hence, it appears that LDs accumulate in these neurons as a result of an inability to clear deposited TAGs, causing a high intracellular concentration of lipids. In our study, the LD content in neurons, but not in microglia, differed between the CA1 and the CA3 regions with neurons of the CA3 having a greater LD content compared with the CA1 neurons. Even within the CA3, we demonstrate the presence of a gradient in the LD content with increasing densities of LDs in pyramidal neurons proximal to the CA2 compared with those close to the DG. The gradient of LDs within the CA3 region of pyramidal cells has never previously been reported, and our findings infer that the regulation of LDs in neurons is not purely a reflection of the levels of available extracellular lipids. Instead, our results highlight that the accumulation of LDs in neurons is regulated in more sophisticated ways, more likely by intrinsic needs related to maintaining plasticity or signaling, or by stressors. Different concentrations or activities of DDHD2 may be an explanation, but this remains to be investigated. Data from the Human Protein Atlas do not indicate that DDHD2 levels differ between the CA1 and the CA3 (DDHD2 protein expression summary - The Human Protein Atlas); however, these data include all cell types in the subregions. Single-cell RNA sequencing from the same source (Single cell type - DDHD2 - The Human Protein Atlas) suggests that principal neurons, both excitatory and inhibitory, are the cells that show the highest expression levels of the *DDHD2* gene, but this study did not separate between different subregions of the hippocampus.

A main difference between the pyramidal neurons of the CA3 and the CA1 is the higher firing rate of the CA1 and richer internal connectivity of the CA3; both of which require a high energy supply, but it is not intuitive whether one of these cell types will have a higher energy demand than the other (36, 37). Pyramidal neurons of the CA1 seem to be more susceptible to oxidative damage and hypoxia (38). If remaining unprocessed in the cytosol, excess fatty acids can be peroxidated and/or cause lipotoxicity, which may lead to ferroptosis. Hence, storage of lipids in LDs may be a way to buffer toxic lipids and protect the neurons (39). A lower number of LDs in pyramidal neurons of the CA1 may represent a lower ability for fatty acid sequestering and protection in these cells compared with the pyramidal neurons of the CA3.

In the present study, we observed that the number of branches per microglial cell was unaffected by the diabetic phenotype or the exercise intervention. A systematic review paper, however, reported that preclinical models of T2DM often showed increased neuroinflammation, including a shift in microglia toward a more ameboid morphology (40). Hence, an unchanged microglial morphology in the present study was somewhat surprising. Our study does not have the statistical power to investigate sex-dependent differences, but all experimental groups consisted of both male and female mice. Previous studies describe an increase in neuroinflammation in T2DM and beneficial effects of exercise as an intervention against diseases involving neuroinflammation (40). Microglia are highly dynamic cells, which can take on a wide range of morphotypes. Recent literature suggests the term “homeostatic” for microglia residing in basal physiological conditions. Similarly, “reactive,” “ameboid,” and “rod” are among the terms suggested for microglia in various reactive states associated with neuroinflammation, disease, or injury. Homeostatic microglia possess small somas and more ramified processes, whereas microglia in more reactive states display larger somas and fewer and less ramified processes (41, 42). Such observations support our measurements of the number of branches, junctions, and end-points as indicators of reactivity. Hyper-ramified microglia represent a transitional state between the homeostatic and the reactive states, but they display increased branching compared with homeostatic cells. Unfortunately, there is no consensus on how to accurately separate the homeostatic and hyper-ramified states through a morphometric inspection alone, and we can therefore not exclude the possibility that for a minority of cells, increased branching was a sign of increased reactivity (42). It should be underlined, however, that microglia in the hyper-ramified state do not necessarily progress into more reactive states; some cells may respond to stimuli with increased branching in a non-harmful way (43, 44).

**Figure S3.**
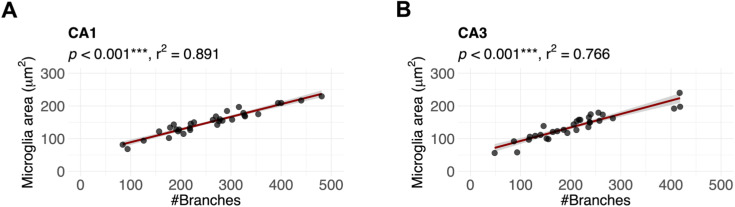
Correlation between the microglial area and degree of microglial activation. Scatter plots. Each dot represents the mean measure from one animal, and the solid line represents the trendline, for which Pearson’s R-value (r2) is given above each plot. The shaded area represents the SD. The x-axis represents the number of branches per microglial cell (#Branches), which is used as a measure for the degree of microglial activation. The y-axis represents the microglial area (μm^2^), and correlation analyses (linear regression models) were performed between these measures and the #Branches (*P*-values for each correlation are given above the corresponding graph). Analyses were performed for all animals combined and corrected for genotype and intervention. **(A, B)** There was a strong positive correlation between the microglial area and the number of branches in both CA1 (A) and CA3 (B). Number of animals (n) for (A): n= 32 mice; (B): n= 30 mice. ***P ≤ 0.001.

Linear regression analyses revealed that a higher density of neuronal LDs was associated with more ramified microglia in the CA1. In contrast, in the CA3, the density of neuronal LDs did not correlate with the degree of microglial activation. Instead, we found that small LD size in neurons correlated with a higher degree of microglial reactivity. The number of LDs per microglial cell did not correlate with the number of branches of the particular microglia. The percentage of microglial area covered by LDs, however, showed a negative correlation to the number of branches (Fig 5E) in the CA1. The latter finding likely reflects a smaller size of less ramified microglia and not an actual increase in LD size or number. This interpretation is supported by the finding that microglial area per se was correlated to the degree of microglial ramification (Fig S3A and B). Previous studies have suggested that the age-related aberrant clearance of synapses, myelin, and cell debris causes microglia to take up excess lipids and store them in the form of LDs (45, 46). Their ability to consume lipid-rich materials correlated to the degree of neuroinflammation, as proinflammatory signals were reported to enhance the internalization of lipids (47, 48). In the present study, no correlation was observed between the LD content of microglia and their degree of reactivity.

Increased LD accumulation in microglia during age-related neurodegenerative disorders has been suggested to represent a decline in the ability of microglia to discard LDs (10, 49) in situations with increased cellular stress. The purpose and consequences of LD accumulation may differ between different cell types. One may speculate whether LDs primarily have a protective role in the brain but may become pathologic with aging or in situations with increased cellular stress (10). Consequently, it is not known whether an increased accumulation of LDs in a specific cell represents an increased need for and/or increased ability to induce LD formation, or, alternatively, an inability to get rid of the neutral lipids stored in this organelle.

Further supporting the notion that microglia in the CA1 and the CA3 subfields of the hippocampus respond differently to external stimuli, we found that LDs in microglia of the CA1 area were smaller in size (Figs 6C and S1B) but more numerous (Fig S1D) in the *db/db* model compared with control mice. The latter comparison did not reach statistical significance when the four groups were analyzed separately (Fig 6E). In the CA3, no differences were observed between the groups when all four groups were compared (Fig 6H–J). Pooling of the data to analyze the effects of the obese/diabetic phenotype regardless of the exercise intervention, however, indicates that although the LD size was not affected (Fig S1F), the number of LDs per microglial cell was increased in the *db/db* mice compared with the *db/*^*+*^ mice (Fig S1H). Based on these known consequences of T2DM in peripheral cells, our finding of reduced LD size in the CA1 and higher LD numbers in the CA1 and the CA3 was intriguing. Elevated circulating lipids have consistently been demonstrated in mouse models of obesity and diabetes, including in the *db/db* model (50), with the expected consequence of increased accumulation of lipids and increased LD density in peripheral organs. Exercise induces an increased release of fatty acids from the adipose tissue leading to a transient increase in free fatty acids in the circulation (51). In the present study, we found that exercise induced a higher number of LDs in the microglia of the CA3, when the data from *db/db* mice and *db/*^*+*^ mice were pooled. Free fatty acids may pass the blood–brain barrier (52). Hence, in response to elevated circulating free fatty acids during exercise, all brain cells would theoretically be exposed to increased free fatty acid concentrations. If these fatty acids are taken up by any brain cell, the cell is expected to produce more LDs to minimize the risk of lipotoxicity. Therefore, the discovered effect of exercise in the CA3 was somewhat expected, but the lack of effect of exercise on LDs in the CA1 was an unexpected observation. The difference in the LD number and size between the CA1 and the CA3 likely reflects a difference in the balance between the uptake of free fatty acids and the rate of β-oxidation in microglia of the two subfields of the hippocampus. In contrast, neither a diabetic phenotype nor exercise affected the LD content in neurons. Because neurons have a very limited capacity for β-oxidation, they cannot easily get rid of fatty acids after import. Hence, these findings suggest that neurons are restricted from taking up free fatty acids, and, unlike most other cell types, do not respond to fluctuations in circulating fatty acid levels by expanding their LD reservoirs.

Accumulation of LDs in microglia has previously been reported in aging and age-related neurodegenerative disorders, and a link to neuroinflammation has been suggested (10, 12). Although free fatty acids may pass the intact blood–brain barrier, TAGs are essentially restricted from passing from blood to the brain (52). In response to aging, obesity (53), and even more so neurodegenerative disorders, the function of the blood–brain barrier is reduced. Seen in connection with the difference in the LD content in neurons of the CA1 and the CA3 of control mice (Figs 2 and 3) and the gradient of neuronal LDs along the CA3 (Fig 4), the lack of effects of a diabetic phenotype and exercise, however, supports the thought that the regulation of LDs in neurons is more sophisticated than just a simple reflection of the availability of lipids.

In the present study, we report that LDs in pyramidal neurons of the hippocampus outnumber the LDs found in the neighboring microglia. Furthermore, there is a difference in the LD content in pyramidal neurons across the hippocampus proper where the largest LD content was found in CA3 pyramidal neurons proximal to the CA2. Whether the differences in LDs reflect differences in energy demand, the need for lipids for membrane remodeling, defense against hypoxia or oxidative stress, or a combination of these and other factors needs further investigation. Our data from the CA1 demonstrate that a higher density of neuronal LDs correlated with microglia of more reactive morphotypes. In the CA3, on the contrary, smaller LD size in neurons correlated with more reactive microglial morphotypes. Neuroinflammation, aging, and neurodegenerative disorders have previously been found to be associated with accumulation of neutral lipids in LDs in microglia. T2DM may cause neuroinflammation and is a risk factor for neurodegenerative disorders. Our results from the *db/db* model demonstrated that T2DM modulates LD dynamics in brain cells: the increased numbers of microglial LDs were observed, but these LDs were smaller in size. The regulatory effect of diabetes was selective to microglia, as LDs in neurons remained unaffected. Exercise was not sufficient to counteract the effects of T2DM, but induced an increase of LDs in microglia in the CA3 subregion. These changes induced by T2DM may partly underlie cerebral effects of this disease and may contribute to a mechanistic link between T2DM, neuroinflammation, and age-related neurodegenerative disorders.

## Materials and Methods

### Animal ethics and housing conditions

All animal experiments were conducted as approved by the Norwegian Food Safety Agency (FOTS ID: #21282) and complied with national and institutional guidelines. These guidelines are in principle equal to ethical guidelines in Directive 2010/63/EU of the European Parliament on the protection of animals used for scientific purposes. Experiments are reported according to the ARRIVE guidelines (54). Mice were housed at the Department of Comparative Medicine at the Faculty of Medicine, University of Oslo, in a room with a stable light/dark cycle (07 AM to 07 PM), with 55 ± 5% relative humidity at 22 ± 2°C in a specific pathogen-free animal unit. Mice were housed in Green Line Sealsafe Plus GM500 or GR900 cages (Techniplast) in groups of up to a maximum of five individuals, depending on cage size. All mice were housed in cages without running wheels, but other environmental enrichment was provided. Mice had ad libitum access to water and chow (62 energy % [E%] of carbohydrate, 11 E% of fat, 27 E% of protein; #RM3A/SDS RM3; Scanbur). In total, 36 mice were used for these studies (19 males and 17 females). One male *db/db* mouse was excluded from the study because of the lack of an obese phenotype. Hence, a total of 35 animals were included in the statistical analysis. The total number of mice per group was estimated based on previous experience with similar experiments (55).

### Animal model

**Figure S4.**
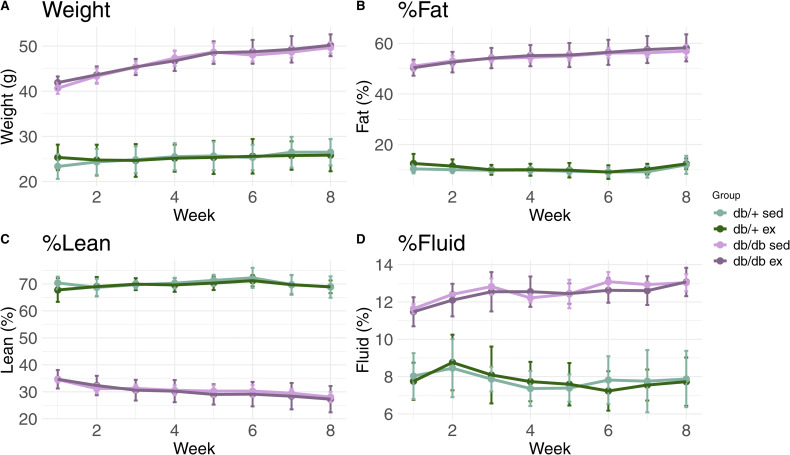
No effects of exercise on body weight or composition. **(A)** Dotted line plots. Mice were weighted (A), and their body composition was measured weekly during their 8-wk high-intensity exercise regimen. **(B, C, D)** Body composition was decided by three factors: fat percentage (B), lean percentage, including soft tissue excluding fat and bones (C), and fluid percentage (D). Mice were grouped into four groups: sedentary *db/*^*+*^ (light green), exercised *db/*^*+*^ (dark green), sedentary *db/db* (pink), and exercised *db/db* (purple). Each point represents the mean weight of the experimental group. Error bars represent the SD.

The strain B6.BKS(D)-Lepr^db^/J, which has a point mutation in the *Lepr* gene that disrupts the function of the leptin receptor, was purchased from The Jackson Laboratory (strain #000697). Because of hyperphagia, homozygote (*db/db*) animals develop an obese and diabetic phenotype from around 8 wk of age on regular chow. Heterozygote (*db/*^*+*^) animals showed no phenotype and were used as healthy controls, as recommended by the supplier. The tissue harvested from ear biopsies was used for genotyping using GenElute Mammalian Genomic DNA Miniprep Kit (G1N350-1KT; Sigma-Aldrich) in accordance with the protocols provided by the manufacturer and The Jackson Laboratory. For the polymerase chain reaction, the One*Taq* Hot Start DNA Polymerase (M0481X; New England Labs) was used in a master mix containing primers (forward outward primer: 5′-TTGTTCCCTTGTTCTTATACCTATTCTGA-3′, reverse outward primer: 5′-CTGTAACAAAATAGGTTCTGACAGCAAC-3′; forward inward primer: 5′-ATTAGAAGATGTTTACATTTTGATGGAAGG-3′, reverse inward primer: 5′-GTCATTCAAACCATAGTTTAGGTTTGTCTA-3′) and target. For animal welfare reasons, their body weights and compositions were monitored throughout the study (Fig S4A–D).

### High-intensity interval exercise regimen

**Figure S5.**
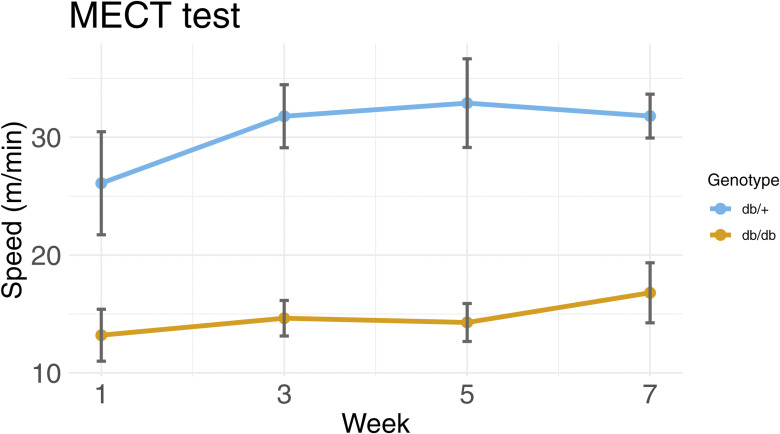
Performance on the MECT by genotypes. Dotted line plots. The maximum endurance test was performed every second week to ensure that the mice were exercising at high intensity. The endurance increased for both groups across the 8-wk exercise regimen. Mice were grouped into two groups: *db/*^*+*^ (blue) and *db/db* (orange). Each point represents the mean speed of the experimental group. Error bars represent the SD.

At 9–11 wk of age, the homozygote (*db/db*) and heterozygote (*db/*^*+*^) mice were randomized to either exercised (ex) or sedentary (sed) groups. From a total of 35 animals, the *db/+* sedentary group consisted of 14 mice (8 males and 6 females), the *db/+* exercised group consisted of 6 mice (3 males and 3 females), the *db/db* sedentary group consisted of 10 mice (5 males, and 5 females), whereas the *db/db* exercised group consisted of 5 mice (2 males and 3 females). The same-sex littermates of both genotypes were housed in the same cage, regardless of whether they were sedentary or received the exercise intervention. Hence, the sedentary mice were brought to the exercise room five times a week together with their exercising littermates. At the time of euthanasia, the mice were 17–19 wk old. The exercise group underwent HIIT for five consecutive days, followed by 2 d of rest per week for 8 wk based on the method described by Morland and colleagues (55), a method optimized to reach ∼90% VO_2max_ and ensure optimal cardiovascular function (55). The HIIT was conducted on treadmills (Columbus Instruments) with a 25-degree incline. The exercise regimen consisted of a 10-min warm-up at a pace of 8 m/min for the *db/*^*+*^ group and a pace of 5 m/min for the *db/db* group, where the difference in pace was an adjustment to the difference in endurance capacity. The maximum endurance capacity of the animals was tested every other week to ensure high-intensity exercise throughout the 8 wk (Fig S5). The maximum endurance capacity test was conducted as described previously (55). The running speed during the HIIT was calculated as 80% of the mean speed reached during the maximum endurance capacity test for all mice that exercised on the same treadmill. Exclusion criteria for the HIIT, set a priori, were as follows: (1) mice that were observed near exhaustion during a HIIT session were given 10-s breaks as needed. (2) If a mouse performed worse than expected (based on the previous performance of the same individual), it would be given additional breaks as needed. If needed, the mouse would be excluded from the rest of the exercise session. If a mouse performed worse than expected for two consecutive exercise sessions, it would be presumed sick or injured, and therefore be withdrawn from the study. (3) Mice that lost weight, experienced fur changes, or displayed stereotypic behaviors were withdrawn from the study. During the study, none of the animals reached criterion 2 or 3.

### Tissue preparation

The tissue for immunohistochemistry was harvested after tissue fixation through transcardial perfusion with formaldehyde. The exercised animals were perfused at 6 h after completing the last session of exercise, and the sedentary mice were perfused directly before or after the exercised mice in the same cage. Deep anesthesia was induced with a mixture of zolazepam (3.3 mg/ml), tiletamine (3.3 mg/ml), xylazine (0.5 mg/ml), and fentanyl (2.6 μg/ml), 10 μl/g administered intraperitoneally (i.p.). A toe pinch test was performed to verify sufficient anesthesia. The fixative (4% PFA in 0.1 M sodium phosphate buffer, pH 7.4) was introduced to the systemic circulation via a cannula inserted into the left ventricle. The cannula was connected to a peristaltic pump, which ensured that the fixative was pumped into the circulatory system at a pace resembling the cardiac output for mice for 8 min. Then, the brains were gently removed from the skull and placed in 4% PFA for 24 h at 4°C before being transferred to and stored in 0.4% PFA at 4°C until cryoprotection and sectioning.

Before cryosectioning, the brains were cryoprotected by immersion in 30% sucrose for 24 h. The brains were cryosectioned into 20-μm-thick sagittal sections with a sliding freezing microtome (HM450; Thermo Fisher Scientific) and placed in 0.1 M NaPi buffer, pH 7.4, containing 0.02% (vol/vol) sodium azide.

### Immunohistochemistry and confocal microscopy

The sagittal sections used in this experiment were selected ∼2.35 mm lateral of bregma, as judged by the neuroanatomy of the hippocampus and the size and shape of the lateral ventricle (Allen Institute for Brain Science, 2008). One free-floating 20-μm-thick brain section from each animal was used for each immunolabeling experiment. These sections were first rinsed two times in PBS, pH 7.4, for 10 min before heat-induced epitope retrieval was performed with 0.1M citrate buffer, pH 8.6, at 80°C for 30 min. The sections were rinsed twice with PBS for 5 min after cooling to room temperature. Then, sections were incubated with blocking solution containing 3% newborn calf serum, 1% BSA, and 0.05% Triton X-100 in PBS for 2 h. Sections used for microglial analysis were incubated with rabbit anti-Iba1 (diluted 1:100, 016-2001; WAKO) and mouse anti-GFAP (diluted 1:500, 3670; Cell Signaling Technologies) in blocking solution overnight at room temperature. The sections were rinsed in PBS 6 times for 10 min before incubation with goat anti-rabbit IgG (H+L) Alexa Fluor 647 nm (diluted 1:500, A27040; Invitrogen) and goat anti-mouse IgG (H+L) Alexa Fluor 594 nm (diluted 1:500, A11032; Invitrogen) for 2 h. The sections were once again rinsed three times in PBS for 10 min before incubation with BODIPY 493/503 (diluted 0.1 mg/ml, D3922; Sigma-Aldrich) in diamidino-2-phenylindole (DMSO) for 30 min before being rinsed with PBS six times for 10 min. Next, the sections were treated with DAPI (diluted 1:5,000 in PBS, D1306; Invitrogen) for 15 min before being rinsed in PBS three times for 10 min. For visualization of neurons, the sections were instead rinsed in PBS three times for 10 min before being incubated with NeuroTrace 530/615 Red (diluted 1:100, N21482; Invitrogen) in PBS for 30 min. The sections were then rinsed in 0.1% PBS with 0.05% Triton X-100 (PBST) for 10 min and two times for 10 min with PBS. The sections were then incubated with BODIPY and DAPI, as described previously.

Images were acquired as Z-stacks (0.3-μm intervals) using a confocal laser scanning microscope (LSM 880 Airyscan; Carl Zeiss). Three Z-stacks (134.10 × 134.10 × 20 μm) were acquired from the CA1 and the CA3 regions of the hippocampus. This represented a mean of 24 microglia/animal and 6552.2 ± 874.6 μm^2^ of the pyramidal cell area. The CA3 region of the hippocampus was imaged starting at the end of the DG and moving along the pyramidal layer toward the CA1, excluding the cornu ammonis 2 (CA2) region by excluding the suspected start of the CA3.

### Image analysis

In each image of a Z-stack, Iba1^+^ and BODIPY^+^ cells were identified using Ilastik software (v.1.3.3) with an algorithm for pixel classification with labels for Iba1 and BODIPY. The images were then further processed for more precise segmentation in the Fiji software (ImageJ, v.2.1.0) plugin Trainable WEKA Segmentation (v.3.3.2). The resulting masks were filtered to remove objects unrelated to microglia. Each microglial cell was counted, and its size (μm^2^) was measured. For the analysis of microglial processes, the Fiji plugins “Skeletonize (2D/3D)” and “Analyze Skeleton (2D/3D)” were used. For LDs in neurons, the CA1 and the CA3 were marked as regions of interest and then extracted from the image in Fiji software, and their area was measured. The color settings were optimized and equaled for all images to avoid bias from manual thresholding. Using the “RedHot” function in “Lookup Tables,” the LDs appeared as circular structures in a white/light orange light spectrum and were easily distinguished from other structures. The image was further processed through the plugin Trainable WEKA Segmentation (v.3.3.2). LDs segmented from neurons were manually verified by the operator. A mask generated by the segmentation was processed using the “Analyze Particles” function to provide a count (number of LDs) and size for LDs. Circular particles between 7 and 200 pixels (equal to 0.5 to 14 μm) in diameter were selected, and comparisons with original images confirmed that these particles corresponded to LDs. The analysis was performed by an observer who was blinded to the genotype and treatment of the animals.

### Statistics

Statistical comparisons between groups were performed in GraphPad Prism version 10. Outliers were excluded if they were more than 1.5 x interquartile range (IQR) below the first quartile or above the third quartile. Normal distribution was tested with Shapiro–Wilk’s test. For data that fulfilled the requirements for normal distribution and equal variance, two-way ANOVA was performed to compare means across more than two groups. One-way mixed-model ANOVA was performed to compare data from more than two dependent samples (for instance, two regions within the same animals). If the ANOVA showed statistical significance, Tukey’s post hoc test was used to assess the significant differences. When comparing two independent groups, the Welch *t* test was performed if the data were normally distributed; for non-normal data, the Mann–Whitney *U* test was performed. In cases where two regions with the same animals were compared, and where equal distribution and equal variance could be assumed, a paired two-tailed *t* test was performed.

Multiple linear regression was performed in R Statistical Software (v4.2.2; R Core Team 2021) (56) using the *lm()* function, where genotype and treatment (exercised/sedentary) were used as covariates. If data did not pass Shapiro–Wilk’s test for normal distribution (*P* ≤ 0.05), the data were log-transformed or square-root–transformed to obtain normal distribution. Hence, three models were computed based on the raw, log-transformed, or square-root–transformed values. The best-fitted model was selected based on the Bayesian information criterion and tested for normality using Shapiro–Wilk’s test. Any significant interference with covariates was further investigated for interactions between slopes of different groups within a given covariate in R using the *emtrends()* function from the emmeans (57) package (v1.8.5) and testing coefficients for interaction with the *pairs()* function from the graphics (56) package (v4.2.2). All plots were generated using GraphPad Prism version 10.

## Supplementary Material

Reviewer comments

## Data Availability

Datasets are available upon request to the corresponding author.
